# A Model of Cancer Stem Cells Derived from Mouse Induced Pluripotent Stem Cells

**DOI:** 10.1371/journal.pone.0033544

**Published:** 2012-04-12

**Authors:** Ling Chen, Tomonari Kasai, Yueguang Li, Yuh Sugii, Guoliang Jin, Masashi Okada, Arun Vaidyanath, Akifumi Mizutani, Ayano Satoh, Takayuki Kudoh, Mary J. C. Hendrix, David S. Salomon, Li Fu, Masaharu Seno

**Affiliations:** 1 Department of Medical and Bioengineering Science, Graduate School of Natural Science and Technology, Okayama University, Okayama, Japan; 2 Japan Society for the Promotion of Science, Tokyo, Japan; 3 Department of Pathology, Tianjin Central Hospital of Gynecology Obstetrics, Tianjin, People's Republic of China; 4 Department of General Surgery, Tianjin 4th Centre Hospital, Tianjin, People's Republic of China; 5 Multidisciplinary Division, Okayama University, Okayama, Japan; 6 Children's Memorial Research Center, Feinberg School of Medicine, Northwestern University, Chicago, Illinois, United States of America; 7 Laboratory of Mammary Biology and Tumorigenesis, Center for Cancer Research, National Cancer Institute, National Institutes of Health, Bethesda, Maryland, United States of America; 8 State Key Laboratory of Breast Cancer Research, Department of Breast Cancer Pathology and Research Laboratory, Cancer Hospital of Tianjin Medical University, Tianjin, People's Republic of China; City of Hope National Medical Center and Beckman Research Institute, United States of America

## Abstract

Cancer stem cells (CSCs) are capable of continuous proliferation and self-renewal and are proposed to play significant roles in oncogenesis, tumor growth, metastasis and cancer recurrence. CSCs are considered derived from normal stem cells affected by the tumor microenvironment although the mechanism of development is not clear yet. In 2007, Yamanaka's group succeeded in generating *Nanog* mouse induced pluripotent stem (miPS) cells, in which green fluorescent protein (GFP) has been inserted into the 5′-untranslated region of the *Nanog* gene. Usually, iPS cells, just like embryonic stem cells, are considered to be induced into progenitor cells, which differentiate into various normal phenotypes depending on the normal niche. We hypothesized that CSCs could be derived from *Nanog* miPS cells in the conditioned culture medium of cancer cell lines, which is a mimic of carcinoma microenvironment. As a result, the Nanog miPS cells treated with the conditioned medium of mouse Lewis lung carcinoma acquired characteristics of CSCs, in that they formed spheroids expressing GFP in suspension culture, and had a high tumorigenicity in Balb/c nude mice exhibiting angiogenesis in vivo. In addition, these iPS-derived CSCs had a capacity of self-renewal and expressed the marker genes, *Nanog*, *Rex1*, *Eras*, *Esg1* and *Cripto*, associated with stem cell properties and an undifferentiated state. Thus we concluded that a model of CSCs was originally developed from miPS cells and proposed the conditioned culture medium of cancer cell lines might perform as niche for producing CSCs. The model of CSCs and the procedure of their establishment will help study the genetic alterations and the secreted factors in the tumor microenvironment which convert miPS cells to CSCs. Furthermore, the identification of potentially bona fide markers of CSCs, which will help the development of novel anti-cancer therapies, might be possible though the CSC model.

## Introduction

A number of studies have attempted to identify the mechanisms underlying malignant tumor growth and progression. Despite significant progress, most therapeutic approaches fail to eliminate all tumor cells. The remaining tumor cells often result in recurrence and metastasis. Recently, the hypothesis of cancer stem cells (CSCs) was proposed to explain the origin of cancer cells. By definition, CSCs are a small fraction of tumor cells with the capacity of both self-renewal and unlimited slow proliferation. They are often resistant to chemotherapy and radiation and thus are responsible for continuously supplying new cancer cells [1]. A current view of the CSCs model is considered that adult stem cells, progenitor cells, or differentiated cells may acquire the multiple genetic and epigenetic alterations required to become CSCs that are involved in promoting and maintaining oncogenesis. This cancer-initiating cell may share some characteristics with adult stem cells residing in the organ, in which they arise, either because organs and tissues originate from resident stem cells or because stem cells are clustered by the properties of their niche [2]. In this context, tumor cells can be epigenetically reverted to tissue specific stem cells when transplanted into a normal stem cell niche [3]–[6].

It is well known that the microenvironment can exert profound genetic or epigenetic effects on stem cells through interactions between cells, or through cell-derived factors originating from the surrounding cells within the niche. These effects can be transient, as seen in the activation of signaling pathways regulating cellular proliferation and migration, or they can be associated with more stable alterations, such as cell fate determination and differentiation [7]. Given the critical role of the microenvironment in cellular regulation, several studies have recently demonstrated that the embryonic stem cell microenvironment could have significant influence on the phenotypic characteristics of aggressive cancer cells [8]–[10]. However, as to whether the tumorigenic microenvironment can affect the fate of stem cells has not been sufficiently explored.

**Figure 1:**
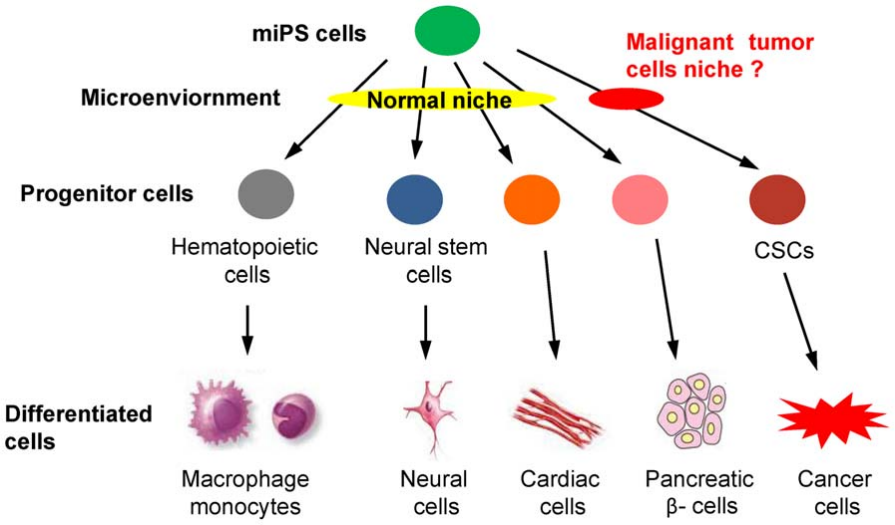
The hypothesis of miPS differentiation when exposed to normal or malignant niche. miPS cells should be induced to some kinds of progenitor cells, such as hematopoietic cells and neural stem cells, differentiating into various phenotypes, such as macrophage, monocytes, neural cells, cardiac cells and pancreatic β- cells, when exposed to the normal niche. We hypothesized that CSCs may also be derived from miPS cells only when exposure to a malignant niche.

In 2007, Yamanaka's group [11] succeeded in generating *Nanog* miPS cells by retroviral transduction of four transcription factors (*octamer 3/4* (*Oct 3/4*), *SRY box-containing gene 2* (*Sox2*), *Kruppel-like factor 4* (*Klf4*) and *C-myc*) into mouse embryonic fibroblasts (MEF). GFP was stably expressed in these cells, but GFP expression was extinguished when these cells were induced to differentiate. To date, miPS cells have been successfully differentiated into various cell types, including hematopoietic and endothelial cells [12], neural cells [13], cardiac cells [14] and pancreatic β-cells [15]. Despite these successful reports of in vitro differentiation, iPS cells are not entirely suitable for transplantation into patients. The main issue is safety concerns in that iPS cells tend to form teratomas and have a risk of malignant transformation [16]–[18]. Based on the CSCs theory, we ascertained whether CSCs can be derived from miPS cells after exposure to a tumor microenvironment (Fig. 1).

## Results

### The miPS cells cultured in the conditioned media of cancer cell lines showed tumorigenicity and angiogensis *in vivo*

**Figure 2:**
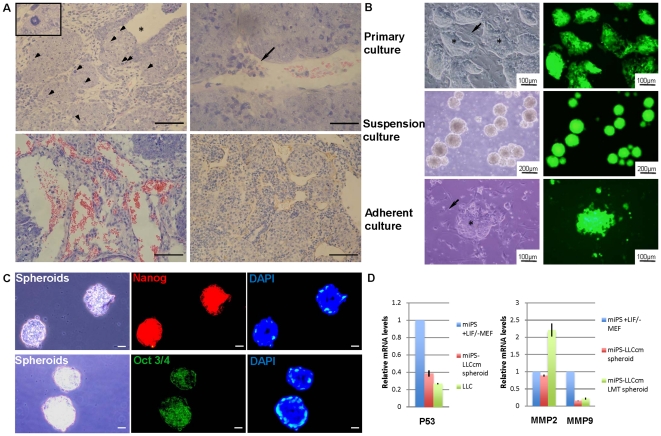
Characterization of miPS-LLCcm cells and derived tumor. (A) Histology of miPS-LLCcm cells derived tumor. The tumor exhibited malignant phenotype with glandular epithelial hyperplasia (asterisk), high nuclear to cytoplasmic ratio, severe nuclear atypia and multiple pathological mitotic figures (arrowheads, inset) (top left); micrometastases (arrow, top right); and hypervascularization indicative of angiogenesis (bottom left) by HE staining. The positive of CD31 (Rat monoclonal antibody, brown) by IHC staining showed multiple vascular vessels in the tumor (bottom right). Scale bars: 100 µm (top left and bottom), 50 µm (top right). (B) Primary culture derived from miPS-LLCcm tumor. The primary culture exhibited stem-like cells (asterisk in top left) expressing GFP (top right) and fibroblast-like cells (arrow in top left) without GFP expression (top right). Spheroid cells grown from the primary culture in suspension (middle left) with GFP expression (middle right). The spheroid cells were placed back in adherent culture maintained stem-like cells (asterisk in bottom left) with GFP expression (bottom right) and fibroblast-like cells (arrow in bottom left) without GFP expression (bottom right). (C) Immunofluorescence staining for Nanog and Oct 3/4 in spheroid cells. Cryosections of spheroid cells were stained with primary antibodies (Rabbit anti-Nanog or Mouse anti-Oct-3/4) followed by anti-Rabbit or anti-mouse secondary antibodies labeled with Alexa fluorophores 555 (red) or 488 (Green). The cells were counterstained with DAPI (blue). Scale bars: 20 µm. (D) The expression levels of p53, MMP-2 and MMP-9 were analyzed by quantitative real-time PCR. miPS+LIF/−MEF, miPS cells cultured with LIF in the medium but without MEFfeeder cells; miPS-LLCcm spheroid, the spheroid cells derived form miPS-LLCcm cells; miPS-LLCcm LMT spheroid, the spheroid cells derived from miPS-LLCcm cells lung metastatic tumor; LLC, Lewis lung carcinoma cells.

In this study, we designed two procedures to treat miPS cells. miPS cells were cultured without feeder cells in a mixture of miPS medium and conditioned medium obtained from the following mouse cancer cell lines: Lewis lung carcinoma (LLC), mouse embryonal carcinoma (P19), mouse melanoma (B16) and mouse mammary carcinoma (MC.E12) for 4 weeks. miPS cells cultured under these conditions were termed miPS-LLCcm, miPS-P19 cm, miPS-B16 cm, and miPS-MC.E12 cm cells respectively. miPS cells were also cocultured with each cancer cell line that had previously been treated with mitomycin C as feeder cells for 4 weeks. These miPS cells were termed miPS-LLCc, miPS-P19c, miPS-B16c and miPS-MC.E12c cells respectively. The miPS cells that had been cultured with or without feeder cells under the different conditions were then transplanted into nude mice. After 4 weeks, miPS cells formed typical teratomas that contained differentiated tissues without metastasis (Fig. S1A). In contrast, mouse allografts of miPS cells that had been treated with conditioned media, miPS-LLCcm, miPS-P19 cm, miPS-B16 cm and miPS-MC.E12 cm cells, formed undifferentiated carcinomas that possessed cells with high nuclear to cytoplasmic ratio, nuclear pleomorphism, aberrantly high mitotic rates, and multiple pathological mitotic figures (Fig. 2A and S1B). On the other hand, only miPS-MC.E12c cells in the coculture group formed malignant tumors (Fig. S1B). The tumorigenicity of the different cells is summarized in Table 1. It is noteworthy that only tumors which were derived from miPS-LLCcm cells showed features of angiogenesis and micrometastases (Fig. 2A).

**Table 1:** Summary of tumorigenic potential of cells derived from miPS cells.

Cell names^a^	Cell number	Tumor formation	Histologic examination
miPS (with feeder cells)	4×10^6^	3/3	Teratoma
miPS (without feeder cells)	4×10^6^	3/3	Teratoma
miPS-LLCcm cells	4×10^6^	8/8	Malignant tumor, angiogenesis
miPS-LLCc cells	–b		
miPS-P19 cm cells	4×10^6^	5/5	Malignant tumor
miPS-P19c cells	4×10^6^	0/3	
miPS-B16 cm cells	4×10^6^	3/5	Malignant tumor
miPS-B16c cells	4×10^6^	0/3	
miPS-MC.E12 cm cells	4×10^6^	3/3	Malignant tumor
miPS-MC.E12c cells	4×10^6^	4/5	Malignant tumor

a : miPS cells were named with each name of cancer derived cells and “c” or “cm”. “c” stands for the miPS cells were cocultured with mouse cancer derived cells treated with mitomycin C and “cm” for the miPS cells cultured in the conditioned medium of cancer derived cells.

b : Cells could not survive after several passages in subculture.

### The miPS-LLCcm cells had a capacity of self-renewal

**Figure 3:**
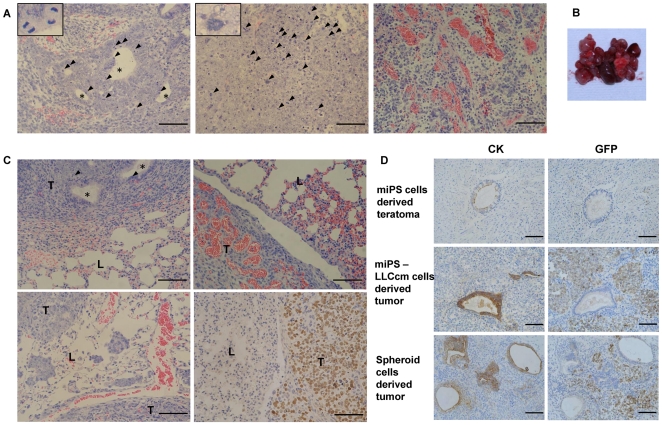
Characterization of tumor derived from spheroid cells. (A) Histology of tumor derived from spheroid cells. The tumor showed some glandular structure (asterisks) with multiple pathologic mitotic figures (arrowheads, inset) (left), high mitotic rates (arrowheads in middle), and hypervascularization (right) by HE staining. Scale bars: 100 µm. (B) Lung metastasis after tail vein injection of spheroid cells. Lungs were occupied by metastatic tumor nodules. (C) The metastases showed some glandular structure (asterisks) with multiple pathologic mitotic figures (arrowheads in top left); hypervascularization (top right); invasion into lung parenchymal tissue (bottom left) by HE staining. The expression of GFP (Rabbit polyclonal antibody, brown) was found in these metastatic nodules by IHC staining (bottom right). T, tumor; L, lung tissue. Scale bars: 100 µm. (D) Immunohistochemistry of CK and GFP localization in tumors derived from miPS, miPS-LLCcm and spheroid cells. Serial sections were stained with CK (mouse monoclonal antibody, brown) and GFP (Rabbit polyclonal antibody, brown), and counterstained with hematoxylin. Glandular region were CK positive but GFP negative in the tumors. Scale bars: 100 µm.

Thirty to fifty percent of the cells were GFP positive in the tumors derived from miPS-LLCcm cells while less than five percent were positive in the differentiated teratoma (Fig. S2). Since GFP was designed under *Nanog* promoter to stably express only in cells which were undifferentiated and would be silenced in differentiated tissues [11], most of miPS cells were considered to be differentiated in the teratomas. On the other hand, the malignant tissues implied to contain undifferentiated stem-like cells. Primary cultures of the tumor should be an effective method to potentially eliminate the differentiated cells in order to obtain more stem-like cells derived from miPS-LLCcm. Thus the tumor tissue derived from miPS-LLCcm cells was subjected to primary culture, from which two distinct types of cell populations were observed. One was stem-like cells that expressed GFP, while the other population was fibroblast-like cells that failed to express GFP (Fig. 2B). Since malignant cells with stem-like properties can be propagated in vitro as nonadherent spheres [19], [20], the cells were transferred to non-adherent culture dishes to facilitate the growth of spheroids. In suspension, GFP expression (Fig. 2B) was observed in these tumor spheres, whereas the fibroblast-like cells could not survive without adhesion to the bottom of dish and was GFP negative. The spheroids derived from miPS-LLCcm tumor were repeatedly trypsinized and confirmed for the capability of forming spheroids under nonadherent condition. Indivisual cells from dissociated spheres were able to form new spheres during serial passage in tissue culture, demonstrating that the cells could self-renew [21]. The tumor spheres were then transferred to adherent culture dishes (Fig. 2B) and were subjected to immunofluorescent staining for Nanog and Oct 3/4 (Fig. 2C). The positive staining of Nanog and Oct 3/4, which are critical factors to sustain the undifferentiated state and self-renewal of stem cells [11], [21], confirmed the expression of the stem cell markers in these spheroids. An aspect of cancerous state of miPS-LLCcm spheroid cells was addressed to the expression of p53 gene by RT-qPCR. As the result, the expression was found downregulated to the level in LLC cancer cells (Fig. 2D). This downregulation may indicate the malignancy of the cells. To evaluate the tumorigenicity of the cells within the tumor spheres, 1×10∼4×10^6^ of these cells were subcutaneously transplanted into nude mice (Table 2). After 4 weeks, tumors formed and exhibited extensive angiogenesis (Fig. 3A), which was similar to the miPS-LLCcm derived tumors. However, these tumors appeared more aggressive due to the high growth rate. To examine the metastatic potential, 1×10^5^ spheroid cells were injected into the mouse tail vein. One month later, multiple metastatic nodules expressing GFP were found in lungs showing that they were derived from spheroid cells (Fig. 3B and 3C). And the expression level of MMP-2 was found significantly upregulated in the spheroid cells derived from miPS-LLCcm cells lung metastatic tumor (miPS-LLCcm LMT spheroid) (Fig. 2D), which implied that miPS-LLCcm cells possess the metastatic potential caused by induction of MMP-2 expression, and the population of highly metastatic cells could be isolated from miPS-LLCcm cells through in vivo panning.

**Table 2:** Summary of tumorigenic potential of miPS-LLCcm spheroid cells.

Cell number	Tumor formation	Histologic examination
1×10	0/4	NA
1×10^2^	0/4	NA
1×10^3^	0/4	NA
1×10^4^	0/4	NA
1×10^5^	2/4	Malignant tumor, angiogenesis
8×10^5^	4/4	Malignant tumor, angiogenesis
2×10^6^	4/4	Malignant tumor, angiogenesis
4×10^6^	4/4	Malignant tumor, angiogenesis

NA: not applicable.

### The tumor derived from miPS-LLCcm cells were composed of adenocarcinomas and abundant undifferentiated tumor cells

We then investigated the type of the malignant tumor by IHC. Pan-Cytokeratin (CK, an epithelial tumor cells marker), vimentin (a marker of mesenchymal tumor), α-actin (a marker of myogenic tumor), CD31 (a marker for vasculogenesis), NF-M and GFAP (markers of neurogenic tumor) were used to stain the tumors (data not shown). CK was found to be strongly stained in the tumors. The expression of CK and GFP was then assessed in multiple serial sections. Glandular regions were CK positive but these cells were GFP negative in the tumors (Fig. 3D). Thirty to fifty percent of the tumor cells were GFP positive in the tumors that had been derived from both miPS-LLCcm cells and primary spheroid cells while no regions were GFP positive in the teratoma. Therefore, these tumors were judged adenocarcinomas mixed with abundant undifferentiated tumor cells.

### The derived cells expressed the embryonic stem cell markers

**Figure 4:**
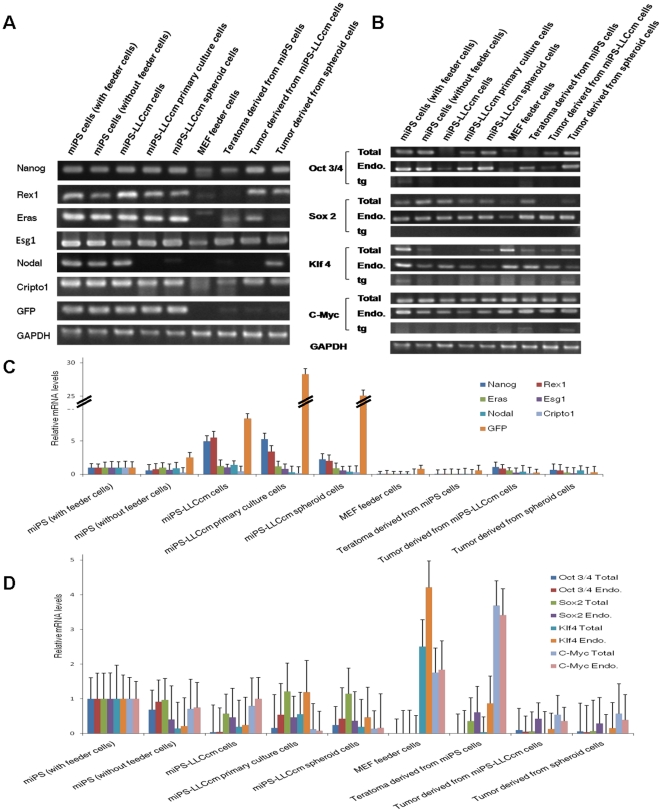
Gene expression in miPS cells, derived cells, MEF cells and tumor tissues. (A) RT-PCR analysis of embryonic stem cell marker gene expression. (B) RT-PCR analysis of the four miPS transcription factors. The PCR products were the coding regions (Total), endogenous transcripts only (Endo.), and transgene transcripts only (tg). (C) Expression levels of embryonic stem cell marker gene were analyzed by quantitative real-time PCR. (D) Expression levels of the four miPS cell transcription factors were analyzed by quantitative real-time PCR.

Embryonic stem cell markers and the four transcription factors that were transduced were then checked by reverse transcription PCR (RT-PCR) and quantitative real-time PCR (RT-qPCR). miPS-LLCcm cells and spheroid cells showed expression of the embryonic stem cell markers (Fig. 4A), but expression levels were somewhat different between these tumor cells and the original miPS cells (Fig. 4C). Specifically, by RT-qPCR *Nanog* and *Rex1* were significantly elevated in the miPS-LLCcm tumor cells, in primary cultures derived from these tumors or in the spheroid cultures as compared to the miPS cells grown in the absence or presence of feeder cells. In contrast, the expression of the four transcription factors was found to be decreased in varying degree in both miPS-LLCcm cells and spheroid cells as compared to the miPS cells that were propagated on feeder cells (Fig. 4B and 4D).

## Discussion

The miPS-LLCcm cells showed spheroid formation in suspension culture, a high tumorigenic potential at limited dilutions and a high metastatic potential, which were all consistent with the basic characteristics of CSCs [19], [22], [23]. Rapid tumor growth requires de novo angiogenesis in which the vascular niche provides growth-promoting signals. The tumors derived from miPS-LLCcm cells including the spheroid cells also showed a high degree of angiogenesis, which was not observed in the teratomas derived from miPS cells. The close association of CSCs and blood vessels has earlier been documented in the nervous system and these vascular niches assist in the maintenance of CSCs [24]. IFNγ, a major negative regulatory molecule of angiogenesis, has been shown to down-regulate the expression of MMPs, inhibit endothelial cell migration as well as induce the angiostatic factor IP-10 through activate of JAK-STAT signal pathway [25]. Microarray assessment comparing the miPS cells and miPS-LLCcm cells showed down-regulation of IFNγR in miPS-LLCcm cells (Materials and Methods S1, Table S1), which might be responsible for the angiogenesis in miPS-LLCcm cells derived tumor. Higher expression of MMP-2 in miPS-LLCcm LMT cells giving the metastatic potential to miPS-LLCcm cells might be a result of IFNγR reduction, although further analysis are required. Taking the downregulation of MMP-9 gene expression in both miPS-LLCcm and miPS-LLCcm LMT cells into consideration, MMP-2 appears responsible for the angiogenesis and metastasis in this study.

Self-renewal is frequently cited as a characteristic of CSCs. However, there are technical limitations to strictly evaluate self-renewal. For normal tissue stem cells, the standard test of self-renewal requires the clonal in vivo demonstration of self-renewal and multi-lineage differentiation in primary transplants of stem cells, followed by demonstration of the same properties in serial transplants of the same cells. Self-renewal in tumorigenic cancer cells has generally been evaluated by the demonstration of serial transplantability of polyclonal tumors and by the demonstration of a similar phenotypic heterogeneity in the parental and progeny tumor xenografts [26]. The sequence of experiments using miPS-LLCcm cells that were derived from primary tumors and their repeated subculture as spheroids demonstrated the capacity of self-renewal in the miPS-LLCcm cells obtaining secondary tumors exhibiting the same histology and phenotype as the primary tumors [21].

Moreover, *Nanog*, a marker widely associated with ‘stemness’ was still expressed at higher levels in the miPS-LLCcm cells and spheroid cells compared to the miPS cells. *Nodal* and *Cripto1* are embryonic morphogens that are responsible for the maintaince of pluripotency/self-renewal in embryonic stem cells and perform a critical role in maintaining the undifferentiated state of cells [7]. In miPS-LLCcm cells, *Nodal* and *Cripto1* expression levels were significantly higher as compared with miPS cells, which confirmed the relatively undifferentiated state of the miPS-LLCcm cells. The decrease of Nodal expression by 70% in spheroid cells probably demonstrated the differentiation of spheroid cells from pluripotent stem cells to unipotent stem cells as CSCs. A significant down-regulation of stem cell markers was observed in teratomas that were derived from miPS cells as these tumors contain mixed populations of different differentiated cell types. In contrast, in tumors derived from miPS-LLCcm cells and spheroid cells, the expression levels of these markers also decreased but remained much higher than those in teratomas. These results, which were consistent with those of IHC, imply that there was a certain amount of the CSCs in the malignant tumors while almost all of the cells were differentiated in the teratomas.

The tumor cells developed in this study from miPS cells grew as spheroids in suspension culture, showed a high tumorigenic and metastatic potential and angiogenesis in vivo. In addition, the capacity for self-renewal and maintainance of an undifferentiated state as assessed by the expression of markers that are associated with embryonic stem cells suggest that these primary miPS-LLCcm cells and the spheroid cells which were derived from miPS-LLCcm cells contain a high proportion of CSCs. Scaffidi and Misteli have recently reported the production of CSC-like cells from fibroblast, which may be traced to somatic stem cells [27]. They transduced oncogenic genes of hTERT, H-Ras V12 and SV40 T antigens to produce CSC-like cells by reprogramming. It should be noteworthy that our CSC model was developed without using gene transduction. This is the first report to demonstrate the development of a CSCs population from miPS cells that can be achieved by factors which are secreted by tumor cells although the identity of these soluble factor(s) remains unknown. Exogenously introduced *C-myc* in the miPS cells may contribute to the transformation since reactivation of *C-myc* carried by a retrovirus was considered to be associated with tumor formation in 20% of chimeric mice [11]. Moreover, leaky expression of these transgenes may also inhibit complete miPS cell differentiation and maturation, leading to a greater risk of immature teratoma formation [28]. However, our results showed that the transgenes that were used to generate miPS cells were almost completely silent in miPS-LLCcm cells and spheroid cells as compared to miPS cells grown on feeder cells and that the two oncogenes, *C-myc* and *Klf4*, were dramatically decreased in the miPS-LLCcm cells and spheroid cells. This suggests that the transgenes may not be the main factors responsible for transformation of the miPS cells in this current model. In addition, because of no tumor formation was observed in the cases of miPS-P19c, -B16c, and of no surviving of miPS-LLCc, which were all in the “co-culture group”, there should be little possibility of the transfer or contribution of mouse tumor viruses in the tumorigenicity of miPS cells cultured in the conditioned medium. Considering the absence of tumorigenicity in these cells in co-culture group, the non-optimal condition of iPS culture should be difficult to explain the conversion of miPS cell whereas the possibility of viral transfection still remains in the cases of miPS-MC.E12 cm and –MC.E12c [29]. Furthermore, four independent works report on genomic analyses of iPS and reveal a worrisome presence of mutations in these cells [30]–[33], which may be cue miPS is easier to be affected by the soluble factor(s) existed in tumor microenvironment. Exosomes are 40–100 nm bilipid membrane vesicles that are secreted by most cell types. They are thought to mediate the cell-cell communication and facilitate biological processes such as cell growth and malignant transformation [34], [35]. Based on the recent reports of tumor exosomes [36], [37], it is worthwhile clarifying the significant role of the exosomes secreted from LLC cells in the conversion of miPS cells to CSCs. Moreover, our finding of downregluration of p53 gene expression in miPS-LLCcm cells indicates that the disturbed p53 network is one of the mechanisms of the conversion of miPS cells to CSCs. It has been reported that tumor cells can inhibit p53 induction in adjacent fibroblast [38]. It is worthwhile noticing that a mechanism of this suppression should depend on the factor secreted from tumor cells, but not on direct cell-to-cell interaction. Definition and characterization of the genetic alterations and the secreted factors in the tumor microenvironment, which convert miPS cells to a CSC will be efficacious for the development of novel anti-cancer therapies.

The expression of specific cell surface markers has been widely used to identify CSCs. Some of these surface markers are known to be common to different CSCs population. However, these markers may still be associated with normal stem cells [1]. Differentially expressed surface markers that can distinguish normal stem cells from CSCs are largely unknown [19]. It is imperative to identify markers that can distinguish between CSCs and normal stem cells. This cellular model in this paper should serve as a viable tool to identify potentially bona fide markers of CSCs. Such markers could be potential targets in the development of novel therapies against CSCs without adversely affecting normal stem cell functions.

## Materials and Methods

### Cell culture

Mouse induced pluripotent stem cells (miPS; cell name: iPS-MEF-Ng-20D-17; Lot No. 012) were purchased from Riken Cell Bank (Japan) and were maintained in medium (DMEM containing 15% FCS, 0.1 mM NEAA, 2 mM L-Glutamine, 0.1 mM 2-mercaptoethanol, 1000 U/ml LIF, 50 U/ml penicillin and 50 U/ml streptomycin) on feeder layers of mitomycin-C-treated mouse embryonic fibroblast (MEF) cells (Reprocell, Japan). Mouse Lewis lung cancer (LLC) cells were purchased from ATCC (USA) and were maintained in DMEM containing 10% FCS; mouse embryonal carcinoma cells (P19) were purchased from Riken Cell Bank (Japan) and were maintained in αMEM containing 10% FCS; mouse melanoma cells (B16/BL6) (ATCC, USA) and mouse mammary tumor cells (BALB-MC.E12) (Riken Cell Bank, Japan) were maintained in MEM containing 10% FCS.

For preparing conditioned medium (CM) from the different mouse cancer cell lines, medium was collected from confluent dishes and filtered using 0.45 µm filter (Millpore, Ireland). Then 3 ml CM were added into 3.5 cm dish overnight to confirm there were no surviving cancer cells in CM. For the conditioned medium experiments, miPS cells (without MEF feeder cells) were maintained in medium described above without LIF. Half of the medium was changed every day with CM for 4 weeks. miPS cells without treatment with CM were used as control. For the coculture experiments, the mouse tumor cell lines were treated with 0.4 µg/ml mitomycin C (Sigma, USA) and were then used as feeder cells and cocultured with miPS cells (without MEF feeder cells) for 4 weeks. miPS cells were passaged every 3 days and cell morphology was photographed using a Olympus IX81 microscope equipped with a light fluorescence device (Olympus, Japan).

For primary culture, mouse allografts were cut into small pieces (approximately 1 mm^3^) in HBSS. After washing three times, the tissues were transferred into a 15 ml tube with 0.25% trypsin of 5–6 fold volume at 37°C for 40 min. Five microliter DMEM containing 10% FCS was then added to terminate digestion. The cellular suspension was then placed into a new tube and centrifuged at 1000 rpm for 10 min. The cell pellet was resuspended in 5 ml HBSS, and centrifuged at 1000 rpm for 5 min. The cell pellet was then placed into an appropriate volume of miPS medium without LIF and the cells were seeded into a dish at a density of 5×10^5^/ml. Cells were passaged every 3 days and cells morphology was observed and photographed using Olympus IX81 microscope equipped with a light fluorescence device (Olympus, Japan).

Suspension cultures to generate spheroids were performed as described in Dontu et al [39]. Briefly, single cells were plated on non-coated dishes (bacterial culture dish) at a density of 2×10^4^/ml in primary culture. Cells were grown in serum-free miPS medium without LIF. Spheroids cells were collected by gentle centrifugation (500 rpm) after 7–10 days and dissociated enzymatically (0.025% trypsin/EDTA).

### Animal experiments

Nude mice (Balb/c Slc*-nu/nu*, female, 6∼8 weeks) were purchased from Charlesriver, Japan. The plan of animal experiments was reviewed and approved by the ethics committee for animal experiments of Okayama University under the IDs OKU-2008211, OKU-2009144, OKU-2010179 and OKU-2011-305.

For transplantation studies, cells (shown in Table 1 and 2) were suspended in 100 µl DMEM containing 10% FCS and injected subcutaneously into nude mice. After 4 weeks, tumors were excised and fixed in 10% neutral formalin buffer solution (Wako, Japan).

For micrometastases studies, 1×10^5^ of miPS-LLCcm spheroid cells were suspended in 100 µl DMEM containing 10% FCS and injected into nude mouse tail vein (n = 6).

### Histologic analysis and immunohistochemistry (IHC)

Tumors were fixed for 24 hours and then processed using a routine wax-embedding procedure for histologic examination. Three micrometer thick sections were stained with hematoxylin and eosin (HE).

IHC for GFP, pan-Cytokeratin, Vimentin, α-Actin, CD31, NF-M, GFAP was performed using formalin-fixed paraffin embedded tissue sections and standard procedures. Briefly, 3 µm tissue sections were deparaffinized and antigen retrieved was performed using microwave exposure at 95°C for 5 minutes in a citrate buffer (pH 6.0) or incubation in proteinase K (40 µg/ml) at 37°C for 30 minutes. After hydrogen peroxide blocking and normal serum blocking (when using mouse monoclonal primary antibody, M.O.M Mouse Ig Blocking Reagent (Vector, USA) as a blocking buffer), the sections were then incubated for 2 h at 37°C with the following primary antibodies: rabbit polyclonal anti-GFP (1∶300, kindly provided by Ayano Satoh, Okayama University, Japan), mouse monoclonal anti-pan-Cytokeratin (AE1/AE3) (1∶200, Santa Cruz, USA), mouse monoclonal anti-Vimentin (1∶200, Santa Cruz, USA), mouse monoclonal anti-α-Actin (1∶200, Santa Cruz, USA), rat monoclonal anti-CD31 (1∶200, Santa Cruz, USA), mouse monoclonal anti-NF-M (1∶50, Santa Cruz, USA), and mouse monoclonal anti-GFAP (1∶200, Santa Cruz, USA). The sections were then incubated with biotinylated anti-rabbit, biotinylated anti-rat or biotinylated anti-mouse secondary antibody (Vector, USA), followed by incubation with the ABC reagent (Vector, USA). Detection was accomplished using 3, 30-diaminobenzidine tetrahydrochloride (DAB, Vector, USA). Incubation of sections with phosphate-buffered saline (PBS) served as negative controls. Counter staining were carried out using hematoxylin.

### Immunofluorescence

The spheroids were fixed in 10% neutral formalin buffer solution (Wako, Japan) for 1 hour and washed in PBS. After centrifugation at 500 rpm during 3 min, the supernatant was removed carefully with a pipette, and then the spheroids were counter-stained with hematoxylin during 30 s. After wash in PBS, spheroids were collected by centrifugation and embedded in OCT compound, and then 6 µm thich cryosections were cut. Cryosections were fixed with 10% neutral formalin buffer solution for 15 min at room temperature, and then incubated with block solution containing 5% BSA, 0.1% Triton X-100 in 0.01 M PBS at room temperature for 1 hour. Sections were then incubated with Rabbit anti-Nanog (1∶100, Abcam, Japan) or mouse anti-Oct-3/4 (1∶100, Santa Cruz, USA) in blocking solution overnight at 4°C. After three washes in PBS, sections were incubated with Goat anti-Rabbit secondary antibodies conjugated to Alexa fluorophores 555 or Goat anti-mouse secondary antibodies conjugated to Alexa fluorophores 488 (1∶400, Invitrogen, USA) for 30 min at room temperature. After three washes in PBS, sections were mounted with Vectashield (mounting medium for fluorescence with DAPI, Vector, USA). Images were acquired using an Olympus IX81 microscope equipped with a light fluorescence device (Olympus, Japan). Sections where the primary antibodies were PBS served as negative controls.

### RNA extraction, RT-PCR and Quantitative real-time PCR (RT-qPCR)

Total RNA from cell lines and tumor tissues were isolated by using RNeasy Mini Kit (QIAGEN, Germany) and TRIzol (Invitrogen, USA), respectively. One µg of total RNA was then reverse transcribed using SuperScript® II Reverse Transcriptase kit (Invitrogen, USA). RT-PCR was performed for 40 cycles for all markers, except *GAPDH* (30 cycles), as follows: denaturing for 2 min at 94°C, annealing for 30 s at 58°C for all primers, extension at 72°C. PCR products were resolved on a 2% agarose gel. Primer sequences were as published in Takahashi et al [40], except *Nodal* (forward primer, 5′ - ATT TGC CAG ACA GAA GCC AAC - 3′, reverse primer, 5′- TCC TCC ACA ATC ATG TCC TTG - 3′), *Cripto 1* (forward primer, 5′ - ATT TGG ACC CGT TGC TGG GAG AGA - 3′, reverse primer, 5′ - CAG CTA GCA TAA AAG TGG TCG TCA - 3′) *p53* (forward primer, 5′ - ACT CTC CTC CCC TCA ATA AGC - 3′, reverse primer, 5′ - TGA TGG TAA GGA TAG GTC GGC - 3′), *MMP-2* (forward primer, 5′ - CAA GTT CCC CGG CGA TGT C - 3′, reverse primer, 5′ - TTC TGG TCA AGG TCA CCT GTC - 3′), *MMP-9* (forward primer, 5′ - CTG GAC AGC CAG ACA CTA AAG - 3′, reverse primer, 5′ - CTC GCG GCA AGT CTT CAG AG - 3′), and *GAPDH* (forward primer, 5′ - CCC TTC ATT GAC CTC AAC TAC - 3′, reverse primer, 5′- CCA CCT TCT TGA TGT CAT CAT - 3′). RT-qPCR was performed with LightCycler 480 SYBR Green I Master (Roche, Germany) according to manufacturer's instructions. Signals were detected with Light Cycler 480 II (Roche, Germany). Amounts of target gene mRNA were normalized to a reference gene *GAPDH*. The primer sequence is same with those of RT-PCR.

## Supporting Information

Figure S1 **Characterization of miPS cells, miPS-P19 cm cells, miPS-B16 cm cells, miPS-MC.E12 cm cells and miPS-MC.E12c cells.** (E) Various tissues present in teratomas derived from miPS cells by HE staining. Scale bars: 100 µm. (F) Histology of miPS-P19 cm cells, miPS-B16 cm cells, miPS-MC.E12 cm cells and miPS-MC.E12c cells derived tumors. The tumors showed malignant phenotype with high nuclear to cytoplasmic ratio, severe nuclear atypia and multiple pathological mitotic figures (arrowhead, inset) by HE staining. Scale bars: 100 µm.
(TIFF)

Figure S2 **IHC of GFP expression.** miPS cell derived teratoma and miPS-LLCcm cell derived tumor were sectioned and stained with anti-GFP antibody (Rabbit polyclonal antibody, brown). Cells were counterstained with hematoxylin. IHC staining, Scale bars: 100 µm.
(TIFF)

Materials and Methods S1 (DOCX)

Table S1 **Genes differentially expressed in miPS-LLCcm cells versus miPS cells.**
(DOCX)
