# Supplementary figures and images for: A Model of Cancer Stem Cells Derived from Mouse Induced Pluripotent Stem Cells
Source: PLoS One. 2012 Apr 12;7(4):e33544. doi: 10.1371/journal.pone.0033544 (PMC3325228; doi:10.1371/journal.pone.0033544)

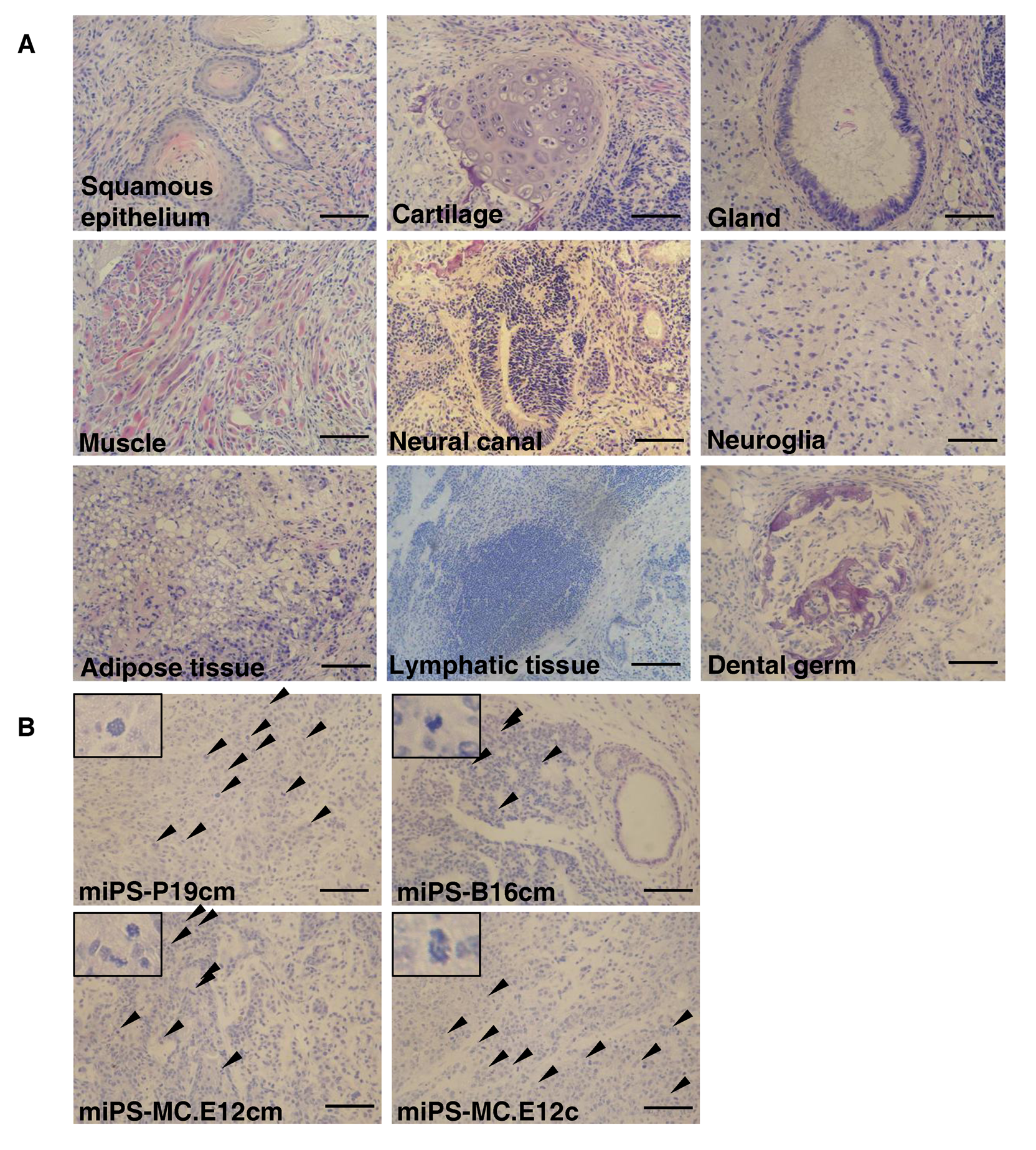

Supplement: Figure S1 — Characterization of miPS cells, miPS-P19 cm cells, miPS-B16 cm cells, miPS-MC.E12 cm cells and miPS-MC.E12c cells. (E) Various tissues present in teratomas derived from miPS cells by HE staining. Scale bars: 100 µm. (F) Histology of miPS-P19 cm cells, miPS-B16 cm cells, miPS-MC.E12 cm cells and miPS-MC.E12c cells derived tumors. The tumors showed malignant phenotype with high nuclear to cytoplasmic ratio, severe nuclear atypia and multiple pathological mitotic figures (arrowhead, inset) by HE staining. Scale bars: 100 µm. (TIFF) [file pone.0033544.s001.tiff]

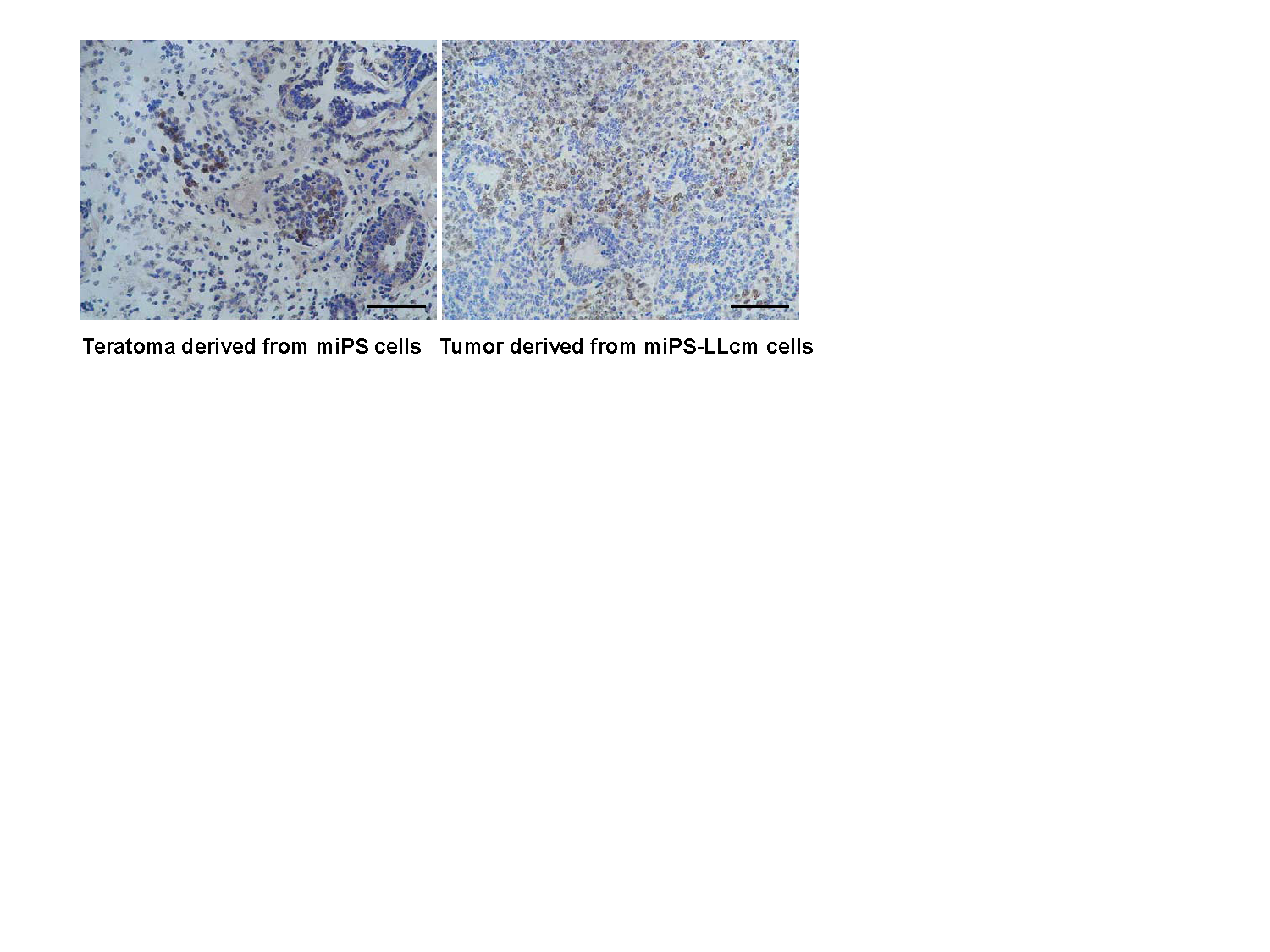

Supplement: Figure S2 — IHC of GFP expression. miPS cell derived teratoma and miPS-LLCcm cell derived tumor were sectioned and stained with anti-GFP antibody (Rabbit polyclonal antibody, brown). Cells were counterstained with hematoxylin. IHC staining, Scale bars: 100 µm. (TIFF) [file pone.0033544.s002.tiff]
