# Supplementary material for: A Model of Cancer Stem Cells Derived from Mouse Induced Pluripotent Stem Cells
Source: PLoS One. 2012 Apr 12;7(4):e33544. doi: 10.1371/journal.pone.0033544 (PMC3325228; doi:10.1371/journal.pone.0033544)
Supplement: Materials and Methods S1 — (DOCX) [file pone.0033544.s003.docx]

**Materials and Methods S1**

**Microarray analysis**

Total RNA isolated from miPS cells and miPS-LLCcm cells were analyzed as described previously [1, 2]. DAN microarray was carrying 1,412-oligonucleotid probes for mouse cell surface proteins. cDNAs were synthesized with Superescript II reverse transcriptase (Invitrogen, USA) with oligo dT primers. Aminoallyl-dUTP was incorporated into cDNAs followed by coupling with Cy-3 dye (Ambion, TX, USA) and was processed for hybridization at 55 °C for 15 hours. The fluorescent images for the hybridization were captured using FLA8000 scanner (Fuji Film, Japan) and analyzed with GenePix Pro5.1 software (Axon Instruments, CA). Gene expression levels were compared to one another by relative fluorescent intensity (RFI), where RFI is the percentage of the fluorescent intensity of each gene and considering that of the internal control to be 100%.

**References**

1. Tuoya, Hirayama K, Nagaoka T, [Yu D](http://www.ncbi.nlm.nih.gov/pubmed?term=%22Yu%20D%22%5BAuthor%5D), [Fukuda T](http://www.ncbi.nlm.nih.gov/pubmed?term=%22Fukuda%20T%22%5BAuthor%5D), et al. (2005) Identification of cell surface marker candidates on SV-T2 cells using DNA microarray on DLC-coated glass. Biochem Biophys Res Commun 334:263-268.
2. Samah AS, Sugii Y, Tuoya, Yu [D](http://www.springerlink.com/content/?Author=Dongwei+Yu), Chen [L](http://www.springerlink.com/content/?Author=Ling+Chen), et al. (2009) Identification of TM9SF2 as a candidate of the cell surface marker common to breast carcinoma cells. Clin Oncol Cancer Res 6: 1-9.
