# Supplementary material for: A Model of Cancer Stem Cells Derived from Mouse Induced Pluripotent Stem Cells
Source: PLoS One. 2012 Apr 12;7(4):e33544. doi: 10.1371/journal.pone.0033544 (PMC3325228; doi:10.1371/journal.pone.0033544)
Supplement: Table S1 — Genes differentially expressed in miPS-LLCcm cells versus miPS cells. (DOCX) [file pone.0033544.s004.docx]

Table S1 Genes differentially expressed in miPS-LLCcm cells *versus* miPS cells

| **Oligo No.** | **Gene** | **Gene full name** |
| --- | --- | --- |
| Genes significantly upregulated in miPS-LLCcm cells | | |
| 591 | Dpep1 | dipeptidase 1 (renal) |
| 1347 | Vnn1 | vanin 1 |
| 598 | Dpagt1 | dolichyl-phosphate (UDP-N-acetylglucosamine) acetylglucosaminephosphotransferase 1 (GlcNAc-1-P transferase) |
| 433 | Alpl | alkaline phosphatase 2, liver |
| 807 | H2-Eb1 | H-2 class II histocompatibility antigen, E-S beta chain |
| 589 | Dsg2 | desmoglein 2 |
| 223 | Htr1d | 5-hydroxytryptamine (serotonin) receptor 1D |
| 505 | Celsr3 | cadherin EGF LAG seven-pass G-type receptor 3 |
| 85 | Dll1 | delta-like 1 homolog (Drosophila) |
| 127 | Fxyd5 | FXYD domain-containing ion transport regulator 5 |
| Genes significantly downregulated in miPS-LLCcm cells | | |
| 268 | Ghr | growth hormone receptor |
| 549 | Clcn7 | chloride channel 7 |
| 402 | Adam1a | A disintegrin and metalloproteinase domain 1a |
| 63 | Sema4b | sema domain, immunoglobulin domain (Ig), transmembrane domain (TM) and short cytoplasmic domain, (semaphorin) 4B |
| 846 | Il1rap | interleukin 1 receptor accessory protein |
| 202 | Il7r | interleukin 7 receptor |
| 348 | F2rl1 | coagulation factor II (thrombin) receptor-like 1 |
| 1038 | Prlr | prolactin receptor |
| 201 | Ifngr1 | interferon gamma receptor |
| 27 | Cd180 | lymphocyte antigen 78 |

Table S1
